# Reversible gating of smart plasmonic molecular traps using thermoresponsive polymers for single-molecule detection

**DOI:** 10.1038/ncomms9797

**Published:** 2015-11-09

**Authors:** Yuanhui Zheng, Alexander H. Soeriyadi, Lorenzo Rosa, Soon Hock Ng, Udo Bach, J. Justin Gooding

**Affiliations:** 1 School of Chemistry, The University of New South Wales, Sydney, New South Wales 2052, Australia; 2 Australian Centre for NanoMedicine, The University of New South Wales, Sydney, New South Wales 2052, Australia; 3 ARC Centre of Excellence in Convergent Bio-Nano Science and Technology, The University of New South Wales, Sydney, New South Wales 2052, Australia; 4 Centre for Micro-Photonics (H34), Swinburne University of Technology, PO Box 218, Hawthorn, Victoria 3122, Australia; 5 Department of Information Engineering, University of Parma, V.le G.P. Usberti 181/A, I-43124 Parma, Italy; 6 Department of Materials Engineering, Monash University, Wellington Road, Clayton, Victoria 3800, Australia; 7 The Melbourne Centre for Nanofabrication, 151 Wellington Road, Clayton, Victoria 3168, Australia

## Abstract

Single-molecule surface-enhanced Raman spectroscopy (SERS) has attracted increasing interest for chemical and biochemical sensing. Many conventional substrates have a broad distribution of SERS enhancements, which compromise reproducibility and result in slow response times for single-molecule detection. Here we report a smart plasmonic sensor that can reversibly trap a single molecule at hotspots for rapid single-molecule detection. The sensor was fabricated through electrostatic self-assembly of gold nanoparticles onto a gold/silica-coated silicon substrate, producing a high yield of uniformly distributed hotspots on the surface. The hotspots were isolated with a monolayer of a thermoresponsive polymer (poly(*N*-isopropylacrylamide)), which act as gates for molecular trapping at the hotspots. The sensor shows not only a good SERS reproducibility but also a capability to repetitively trap and release molecules for single-molecular sensing. The single-molecule sensitivity is experimentally verified using SERS spectral blinking and bianalyte methods.

## Abstract (web summary)

Conventional substrates used for surface-enhanced Raman spectroscopy (SERS) are slow in response and lack reproducibility. Here, Zheng *et al.* describe a plasmonic sensor that can trap a single molecule at hot spots for rapid single-molecule detection with repeated trap and release capability and good SERS reproducibility.

## Graphical abstract

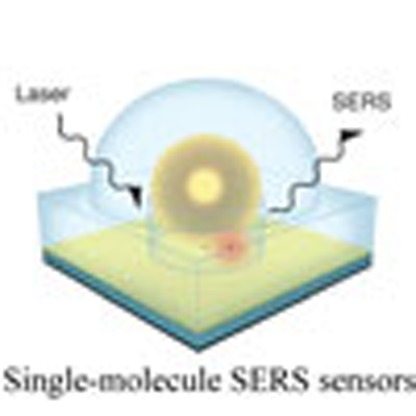

Surface-enhanced Raman spectroscopy (SERS) is one of the few techniques that are capable of detecting and identifying chemical and biological compounds with single-molecule sensitivity^1^,^2^,^3^,^4^,^5^,^6^. This technique takes advantage of plasmonic (metal) nanostructures to amplify Raman signals. A unique feature of these metal nanostructures is they show a resonant oscillation of their conduction electrons on light irradiation. This light-matter interaction leads to an enormous electromagnetic field enhancement in the close vicinity of the metal surfaces. The field enhancement is particularly strong at sharp corners or tips^1^,^7^, interparticle gaps^8^,^9^,^10^,^11^,^12^,^13^ and nanoscale pores^4^,^14^ typically referred to as ‘hotspots'. Although the importance of hotspots has been both experimentally and theoretically demonstrated for SERS sensing^1^,^7^,^8^,^9^,^10^,^11^,^12^,^13^,^14^, the fraction of analytes adsorbed to the hotspots for a conventional SERS substrate is extremely small due to the low spatial occupation of hotspots per unit area^14^,^15^. For example, a silver film-over-nanosphere SERS substrate showed a wide distribution of SERS enhancement factors (EFs) ranging from 2.8 × 10^4^ to **>**1 × 10^10^ (ref. 15). Yet, the hottest spots, with SERS EFs larger than 10^9^, only accounted for 63 out of a million of total Raman-active sites^15^. There is therefore a prevailing need for the development of innovative SERS substrates that have a large number of uniformly distributed hotspots and the analyte molecules can be confined only at the hotspots.

Several concepts have been developed with the aim to adsorb target analytes only at the hotspots^16^. The most straightforward one is the isolation of hotspots with a chemically inert material. Diebold *et al.*^17^ developed a near-field optical lithography method to isolate hotspots on a macroscopic SERS substrate composed of an array of nanocones covered by a thin layer of a photoresist. The excitation of the nanocones with a laser scanning across the substrate results in a strong near field at the tips of the cones (that is, hotspots), which causes preferential exposure of the photoresist at the hotspots. The removal of the exposed photoresist yields a substrate for which only the hotspots are available as binding sites. A requirement for detection in such a sensor however is the analytes having a strong affinity for the metal. A promising alternative approach is the analyte trapping at hotspots. Hu *et al.*^18^ demonstrated a molecular trap based on gold-coated flexible polymer fingers for SERS sensing. The tips of these gold nanofingers were brought together by the capillary force of solvent evaporation, resulting in molecules trapped between the tips^18^. This drying process inevitably results in the deposition of analytes outside the hotspots. Álvarez-Puebla *et al.*^19^ developed a more controllable trapping system made of microgels. These microgels are composed of stimuli-responsive polymer-coated gold nanoparticles (AuNPs). The polymer shell either swelled or collapsed when responding to the external temperature. This change in volume was utilized as a means to trap the analytes and get them close to the metal surface, where the electromagnetic field is significantly enhanced. However, the overall SERS enhancement from these individual colloidal nanoparticles (NPs) is usually insufficient for single-molecule detection. To date, many complex plasmonic nanostructures, such as film-coupled metallic NPs (also referred as to NPs-on-mirror)^20^,^21^,^22^,^23^,^24^,^25^,^26^,^27^,^28^, metal NP assemblies^2^,^3^,^6^,^7^,^8^,^9^,^10^,^11^,^12^ and porous metal films^4^,^14^ have been fabricated for SERS applications. Among all of these, film-coupled metallic NPs are of special interest for two reasons. First, the simplicity of this system makes it an ideal model for theoretical simulation studies^24^,^25^,^26^,^27^. Second, it has been shown that such a system enables SERS-based single-molecule detection^23^.

In this work, we develop a smart plasmonic molecular trap based on a well-established film-coupled AuNP system on a silica-coated silicon optical interference substrate and demonstrate a gating mechanism to control the trapping and release of analytes at the particle–substrate gaps (that is, hotspots) for SERS-based single-molecule detection. Silica-coated silicon substrates are chosen as the silica layer can generate an additional SERS enhancement up to 50 times due to an interference effect^29^. The hotspots of the molecular trap developed here are isolated with a self-assembled monolayer of thermoresponsive polymer, which acts as gates for the reversible molecular trapping at the hotspots. The trapped molecules can be subsequently released after SERS sensing. This reversible trapping process makes it possible to detect an abundance of analytes in one measurement but also to reuse the SERS substrate multiple times.

## Results

### Sensor fabrication

**Figure 1:**
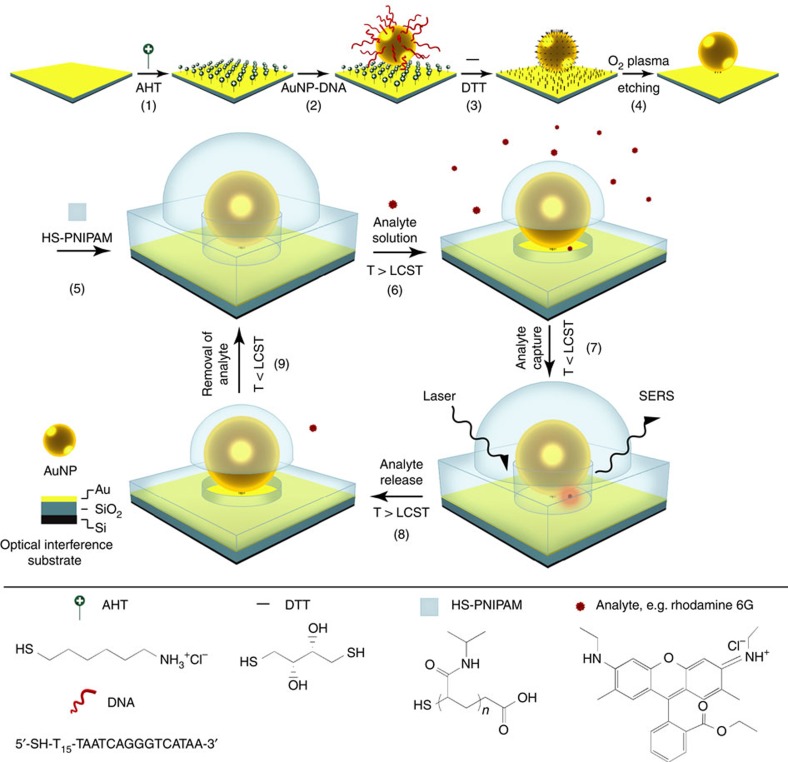
Fabrication and sensing mechanism. An optical interference substrate, composed of 15 nm gold/110 nm silica on a silicon wafer, is modified with a monolayer of AHT (step 1). The AHT functionalized substrate is exposed to a solution of DNA-AuNPs to allow for their electrostatic adsorption (step 2). The DNA and AHT are then displaced with DTT (step 3). The DTT molecules outside the particle–substrate gap (that is, hotspot region) are selectively removed by oxygen plasma etching (step 4). Following the oxygen plasma treatment, the substrate is exposed to an ethanolic solution of HS-PNIPAM to allow for the formation of self-assembled monolayer on the AuNP and the gold film, isolating the hotspots (step 5). For SERS sensing, the substrate is exposed to an analyte (for example, rhodamine 6G) solution with a temperature (50 °C) higher than the LCST (∼34.5 °C) of the polymer. This temperature triggers the shrinkage of the polymers to allow the analyte molecules to flow into the molecular traps (step 6). Subsequently, the substrate is cooled down to a temperature (4 °C) much smaller than the LCST of the polymer. In this case, the polymer shell expands and the analyte molecules are trapped at the hotspots (step 7). Excess analyte molecules are removed by washing with a cold (4 °C) aqueous solution before the SERS measurements. After the SERS measurements, the substrate is exposed to a hot (50 °C) aqueous solution to release the analyte molecules (step 8) and then separated from the solution by disposing of the solution (step 9).

The fabrication of the smart plasmonic molecular traps and their SERS sensing mechanism are schematically illustrated in Fig. 1. Gold/silica-coated silicon substrates were fabricated by evaporation of a 15-nm gold film on a 110-nm silica-coated silicon wafer using 3-mercaptopropyltrimethoxysilane as an adhesion layer. A freshly prepared gold/silica-coated silicon optical interference substrate was exposed to an ethanolic solution of 6-amino-1-hexanethiol (AHT) to form a self-assembled monolayer on the gold film (step 1), which confers a net positive surface charge on the substrate at neutral pH^12^,^30^. Commercially available spherical AuNPs (average diameter: 80 nm) functionalized with monothiolated DNA (referred to as DNA-AuNPs) were used as building blocks to produce an array of well-spaced NPs on the AHT-modified substrate. Exposing the negatively charged DNA-AuNPs to the AHT-modified substrate resulted in NP adsorption on the substrate driven by electrostatic attractions between the particles and the substrate (step 2). The strong repulsive electrostatic forces between DNA-AuNPs predetermine their separation during the assembly. These forces ultimately rely on parameters such as the AuNP concentration, surface charge density and the ionic strength of the medium^11^. All of these can be experimentally controlled. This allows us to achieve high levels of surface coverage of AuNPs on the substrate and minimize the distance between the neighbouring particles to avoid their surface plasmon coupling. Once the AuNP array is formed, the substrate was exposed to a dithiothreitol (DTT) aqueous solution. This results in the binding of the AuNPs to the underlying gold film and displacement of the DNA and AHT with DTT (step 3). The DTT molecules outside the particle–substrate gaps are selectively removed by oxygen plasma etching (step 4).

Following the oxygen plasma treatment, the substrate was exposed to an ethanolic solution of thiolated poly(*N*-isopropyl acrylamide) (HS-PNIPAM, *M*_w_=4.7 × 10^4^ g mol^−1^) to allow for the adsorption of PNIPAM on the AuNP and the gold film surfaces via thiol-gold bonds (step 5). The thiolated PNIPAM used here was synthesized according to the method described by Wong *et al.*^31^ (Supplementary Methods) and its lower critical solution temperature (LCST) is determined to be ∼34.5 °C (Supplementary Fig. 1). The PNIPAM is in an extended conformation in the ethanolic solution^32^ and cannot adsorb to the particle–substrate gap due to the steric hindrance, yielding molecular traps with SERS hotspots isolated. For SERS sensing, the molecular traps were exposed to an analyte, for example, rhodamine 6G (R6G), solution at a temperature (50 °C) higher than the LCST. The high temperature induces the shrinkage of the polymers, allowing the analyte solution to flow into the molecular traps (step 6). Subsequently, the molecular traps were cooled down to a temperature (4 °C) that is lower than the LCST. At this temperature, the polymers expand to its original conformation and the analyte molecules are captured in molecular traps (step 7). During the analyte trapping process, an oxalic acid solution is used to adjust the pH of the analyte solution to 2, where very few of the carboxyl groups on the polymer are deprotonated^16^. This minimizes the nonspecific adsorption of the analyte on the polymer shell through electrostatic interaction. The non-adsorbed analyte molecules were removed by washing with the cold oxalic acid solution. Thereafter the sample spontaneously dried in air at room temperature (∼25 °C) when being taken out from the cold oxalic acid solution. This process happens within a few seconds. During this drying process, the thermoresponsive polymers remain in the extended conformation, as the polymer's LCST is much higher than the room temperature. When drying in air, the trapped analyte molecules are drawn to the centre (the hottest region) of the molecular traps by the capillary force of the solvent evaporation^33^. The molecular traps are then ready for the SERS measurements. Following the SERS measurements, the molecular traps were exposed to a hot (50 °C) oxalic acid solution to release the analytes (step 8) and then separated from the solution (step 9). After the analyte molecules were released, the molecular traps are ready for next cycle of Raman spectroscopic analysis.

### Sensor characterization and near-field simulation

**Figure 2:**
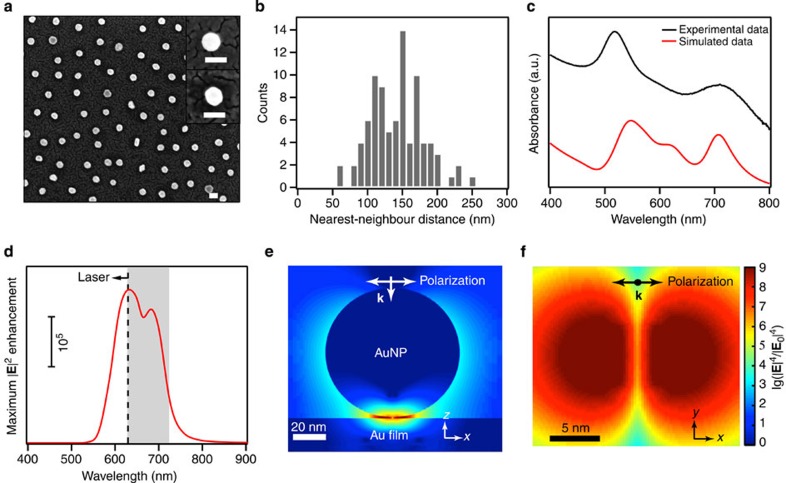
Characterization and simulations. (**a**) A typical scanning electron microscopy micrograph of an array of 80 nm AuNPs on a gold film covered with a monolayer of HS-PNIPAM (scale bar, 100 nm). The insets are of a AuNP on gold film before (upper) and after (lower) surface modification with the HS-PNIPAM. (**b**) Distance analysis of the self-assembled AuNPs: the distance between each AuNP and its nearest neighbour was measured (edge to edge) using image analysis. (**c**) Absorption spectra of the AuNPs on gold film (experimental: black line; calculated: red line). (**d**) Calculated maximum field intensity enhancement at the hotspot as a function of wavelength in the range of 400–900 nm (particle–substrate gap: 0.7 nm). The dash line and grey shadow area show the laser wavelength and Raman shift region of interest, respectively. (**e**,**f**) Simulated spatial SERS enhancement factor (|**E**|^4^/|**E**_0_|^4^) distributions at a 80-nm AuNP–15-nm Au film junction sampled along the planes vertical (*xz*) and horizontal (*xy*) to the sample plane (*xy*), respectively.

Figure 2a shows a typical scanning electron microscopy image of the produced AuNP array on a gold/silica-coated silicon substrate. As shown in Fig. 2a, all particles are coated with PNIPAM as indicated by the darker ring (that is, polymer shell) around the particles (see inset). The observation of carbon, nitrogen and sulphur signals from the sample by X-ray photoelectron spectroscopy further confirms the presence of PNIPAM on the gold surfaces (Supplementary Fig. 2). The atomic percentages of these three elements are listed in Supplementary Table 1. The C–C/C–N/O=C–N ratio derived from the X-ray photoelectron spectroscopy measurement is 4.3:1:1.1, which is in good agreement with the theoretical value (4:1:1). The particle density and the polymer shell thickness are estimated to be ∼14 particles per μm^2^ and ∼50 nm, respectively. The distance between each AuNP and its nearest neighbour was determined via image analysis. The statistical analysis (Fig. 2b) shows an average nearest-neighbour distance of 138 nm with a s.d. of 38 nm. At such separation distances there is no coupling between particles. The optical property of the AuNP arrays on gold-coated glass substrates was recorded using ultraviolet–visible absorption spectroscopy (Fig. 2c, black line). The sample shows two distinct surface plasmon resonance peaks at 520 and 710 nm, which are ascribed to the dipole surface plasmon resonances parallel and perpendicular to the gold film, respectively^24^,^25^,^26^,^27^,^28^. To describe semi-quantitatively the film-coupled spheres, we simulated their absorption spectrum and electric field enhancement using three-dimensional finite-difference time-domain method. By carefully adjusting particle–substrate distance in our modelling (Supplementary Fig. 3), we were able to reproduce the qualitative features of the measured absorption spectrum (Fig. 2c, red line). From the simulations, the particle–substrate distance is estimated to be ∼0.7 nm, which is slightly smaller than the length of DTT molecules (∼1.0 nm). This suggests some molecular rearrangement within the gap between the nanoparticle and the underlying gold substrate^34^. Figure 2d shows the maximum local electromagnetic field intensity enhancement in the nanogap region (gap size: 0.7 nm) with respect to source intensity. It can be clearly seen that the enhancement mainly occurs in the range of 530–800 nm. To achieve maximum SERS enhancement, we chose 633-nm laser as an excitation source (dash line) and a typical Raman spectrum (0–2,000 cm^−1^, grey shadow) falls in the maximum enhancement region. Figure 2e,f show the spatial SERS enhancement factor (|**E**|^4^/|**E**_0_|^4^) distributions of a single-film-coupled sphere at the excitation wavelength of 633 nm. It is clear that the field enhancement is localized in the gap between the particle and the gold film. The average SERS EFs originated from near-field coupling is estimated to be ∼10^9^ at the hotspot using the SERS EF boundary criterion of 10^7^. The corresponding hotspot volume is calculated to be 48 nm^3^ (Supplementary Fig. 3 and Supplementary Table 2).

### Reversible molecular trapping and high SERS reproducibility

One application of the smart plasmonic molecular traps is molecular sensing based on surface-enhanced Raman signals at the hotspots. R6G, one of the most widely used Raman-active dyes, has a maximum of absorption at 545 nm and almost drops to zero at a wavelength higher than 600 nm (Supplementary Fig. 4). It therefore can be considered as a non-resonant dye at 633 nm excitation^6^. Previous studies have shown that a SERS EF of ∼10^7^ is sufficient to detect single R6G molecules adsorbed on AgNP aggregates at 633 nm laser excitation^6^. As discussed earlier, the smart molecular traps developed here exhibit a high average SERS EF of ∼10^9^, which allows them to detect single molecules. To demonstrate this potential, we investigate their SERS activity using R6G as a model analyte.

**Figure 3:**
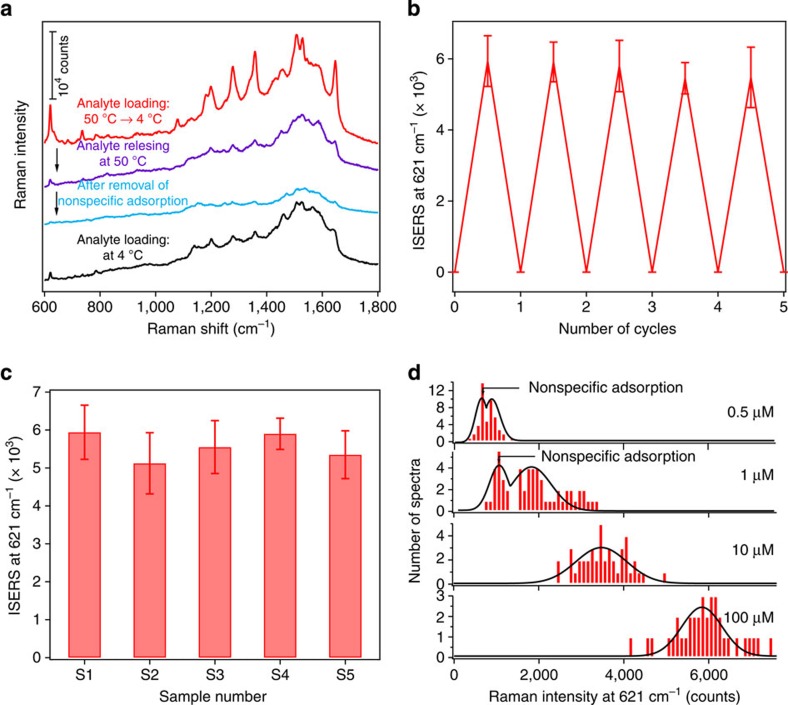
SERS performance using rhodamine 6G as a model analyte. (**a**) SERS activity at the different stages of the sensing scheme illustrated in Fig. 1: analyte trapped (red curve); analyte released (purple curve); and nonspecific adsorption removed (blue curve). For comparison, a control experiment with analyte loading at 4 °C (black curve) is provided in **a**. (**b**) Cycling SERS activity, (**c**) substrate-to-substrate SERS intensity variation at 621 cm^−1^ measured for five different substrates (each intensity value represents the average of 10 measurements at different spots and the s.d.'s for each sample are shown as error bars) and (**d**) analyte concentration-dependent SERS activity. The analyte-loading concentration for the samples shown in **a**–**c** is 100 μM. For all samples, *λ*_ex_=633 nm, *P*_ex_≈3.5 mW, acquisition time= 10 s and laser spot size≈2 μm^2^.

Figure 3a shows the SERS activity of the smart molecular traps at the different stages of the sensing scheme illustrated in Fig. 1. All of the spectra were obtained at 633 nm laser excitation. Prominent Raman modes at 621, 1,200, 1,280, 1,360, 1,510 and 1,642 cm^−1^ originated from R6G^5^,^6^,^35^ are observed (red line), when the molecular trap was exposed to a 100-μM R6G solution at 50 °C for 3 min and then cooled down to 4 °C (see Approach 1 in Methods for the experimental details). The exposure of the molecular trap to the high temperature causes the polymer shells to collapse, and thus the analyte solution flowing into the molecular traps. Subsequent cooling of the solution brings the polymer chains back to its original the extended conformation, resulting in analyte molecules trapped. The trapped molecules are brought to the hotspot region with the SERS EFs of >10^7^ driven by the capillary force of solvent evaporation. Further exposure to a hot oxalic acid solution leads to the shrinkage of the polymer shells and the release of the trapped molecules from the hotspots. This results in the marked decrease of Raman signals (purple line). These weak Raman signals are ascribed to the nonspecifically adsorbed R6G molecules on the polymer shells due to electrostatic or hydrophobic–hydrophobic interactions between the analyte and the polymer, as they were also observed when the molecular trap was exposed to the analyte solution at 4 °C (black line). This nonspecific adsorption can be completely removed by washing with a mixture of water and methanol (blue line). After the complete removal of the nonspecific adsorption, the molecular trap is ready for the next cycle of sensing process. Figure 3b shows the cyclic sensing capability and reusability of the molecular trap. Similar SERS intensities at the Raman peak of 621 cm^−1^ are observed each time when the molecular trap was subjected to a total of five consecutive sensing cycles.

Good reproducibility and high sensitivity are two key requirements for an ideal SERS sensor. We therefore undertook a statistical analysis to quantify the variation in the SERS signal intensity between different locations on one substrate (spot-to-spot variation) and between different substrates (substrate-to-substrate variation). Figure 3c shows the spot-to-spot Raman intensity variation of five sensors that resulted from independent fabrication process shown in Fig. 1. For each sensor the 621 cm^−1^ Raman peak height was measured at 10 different spots. The highest spot-to-spot coefficient of variation among the five samples is 15.7% and the substrate-to-substrate coefficient of variation is about 5.6%. Such a good reproducibility indicates that the self-assembly process presented here provides excellent control over the particle density and the particle–substrate distance. Furthermore, we also evaluated the detection limit of the sensor. Figure 3d shows the Raman intensity of R6G at 621 cm^−1^ as a function of its concentration. As expected, the Raman intensity decreases with decreasing R6G loading concentration. At an elevated R6G concentration, for example, 10 and 100 μM, many molecules are trapped at hotspots. At the same concentration, R6G can also nonspecifically adsorb on the areas outside the hotspots. The observed SERS signals are from both the trapped and the nonspecifically adsorbed molecules. When decreasing R6G concentration to a point where single-molecule trapping is reached, from statistical analysis point of view, there are areas with no analytes trapped in hotspots but minor amounts of nonspecific adsorption may still occur. This inevitably results in two distribution peaks of Raman intensity. As shown in Fig. 3d, two distribution peaks of Raman intensity at 621 cm^−1^ are observed at R6G concentration of 1 μM. The one at the lower intensity is attributed to nonspecific adsorptions, while the other one at higher intensity predominantly stems from the trapped molecules at hotspots. On the basis of the particle density, the volume of a single-molecular trap and the size of the laser spot, the number of analyte molecules trapped in hotspots within the observation area is estimated to be ∼1.1 molecules when the analyte concentration is 1 μM (Supplementary Fig. 5 and Supplementary Note 1). Further decreasing of the analyte concentration creates a new situation where there are areas having either trapped molecules in hotspots or nonspecific adsorptions. Meanwhile there are areas having neither trapped molecules in hotspots nor nonspecific adsorptions. This explains why we also observed two distribution peaks of SERS intensity at R6G concentration of 0.5 μM. Interestingly, the SERS intensity of the second distribution peak at R6G concentration of 0.5 μM is close to the intensity difference between the two distribution peaks at R6G concentration of 1 μM. This indicates that the first SERS intensity distribution peak at R6G concentration of 0.5 μM is related to nonspecific adsorptions, while the second one corresponds to single-molecule SERS.

### Single-molecule SERS blinking and detection of bianalytes

**Figure 4:**
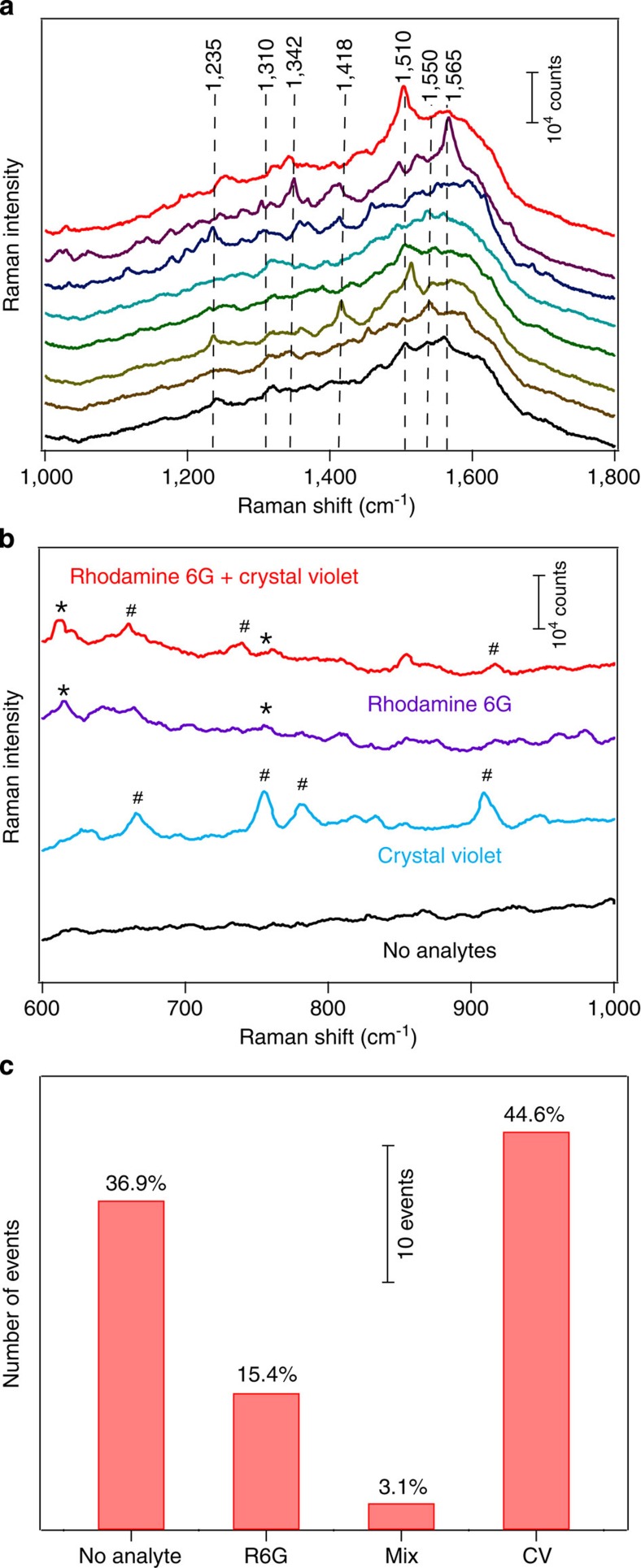
Single-molecule behaviours from the smart molecular trap. (**a**) Single-molecule blinking SERS spectra of R6G captured at the concentration of 1 μM, (**b**) single-molecule SERS detection of bianalytes: four representative SERS spectra showing no analytes (black curve); a pure CV event (blue curve); a pure R6G event (purple curve); and a mixed event (red curve), and (**c**) histogram of occurrences of none, pure R6G, pure CV and mixed molecules from 65 different spots of a molecular trap. The concentration of the two analytes is 0.5 μM, respectively. SERS measurement conditions: *λ*_ex_=633 nm; *P*_ex_≈3.5 mW; acquisition time=10 s; and laser spot size≈2 μm^2^.

Several single-molecule SERS verification experiments including Poisson distribution of intensities^36^,^37^, Raman spectral blinking^2^,^38^,^39^,^40^ and bianalyte approach^37^,^41^,^42^,^43^ have been developed. To further confirm single-molecule sensitivity, we conducted a time-dependent SERS experiment and a bianalyte experiment (see Approach 2 in Methods for the experimental details). The time-dependent SERS experiment was carried out by repetitively measuring the SERS spectra of R6G from the same spot of the molecular trap (R6G loading concentration: 1 μM). Figure 4a shows that the Raman peaks of R6G randomly appear and then disappear during the SERS measurements at the same location. This spectral blinking phenomenon has not been observed at higher concentration of R6G and is considered as a characteristic of the behaviour of single, or a few, molecules^2^,^38^,^39^,^40^. For the bianalyte experiment, we used R6G and crystal violet (CV) as the model analytes. Their concentration in the mixture is controlled to be 0.5 μM, respectively. This concentration was chosen to ensure that approximately one molecule is trapped in each probe region (laser spot) on the substrate based on our estimate of the number of molecular traps (Supplementary Fig. 5 and Supplementary Note 1). Typical SERS spectra from four different spots are shown in Fig. 4b. Two of the spectra (blue and purple curves) show the typical fingerprint peaks of CV^44^ and R6G, respectively (see Supplementary Fig. 6 for peak assignments). For a series of SERS measurements at 65 different spots, we observed that the SERS spectra were dominated by either one analyte (CV: 44.6%) or the other (R6G: 15.4%), or no molecules (36.9%) at all (Fig. 4c). Only 3.1% of measurements showed a mixed spectrum (Fig. 4b, red curve). Since both R6G and CV do not have specific affinity for the gold surface, they should have similar probability of being captured at the molecular traps. However, the statistical analysis of single-molecule events shows that CV has ∼3 times higher probability to be present in hotspots than R6G (Fig. 4c). This indicates that CV may have stronger physicochemical affinity to the gold surface than R6G. The bianalyte results shown in Fig. 4b,c are in good agreement with previous reports providing evidence for single-molecule SERS^37^,^41^,^42^,^43^. The ability to trap and detect single molecules in the micromolar range, where the majority of biomolecular interactions and enzymatic activity takes place, would allow us to exploit its potential applications in diagnostics and biosensing^45^. This requires the further development of new smart polymers that can respond to various environmental (for example, temperature, pH and light, etc.) changes^46^ and show excellent biocompatibility and antifouling property^47^. This work is under way.

## Discussion

In conclusion, we have developed a smart plasmonic sensor that consists of spherical AuNPs on a gold/silica-coated silicon optical interference substrate. The sensor is fabricated through electrostatic self-assembly of AuNPs onto the optical interference substrate. The electrostatic self-assembly strategy developed here is particularly advantageous in terms of achieving a high AuNP density and maintaining a minimum interparticle distance to avoid surface plasmon coupling between the neighbouring particles. The formed particle–substrate gaps are isolated with a self-assembled monolayer of a thiolated PNIPAM, which exhibits reversible conformational changes in response to temperature. The polymer shell acts as gates for molecular trapping at the hotspots that show an exceptionally high average SERS EF of ∼10^9^ calculated using the SERS EF boundary criterion of 10^7^. The reversible conformational change of the polymer shell makes it possible to reuse the sensor multiple times. The produced sensor also shows an excellent SERS reproducibility as well as an ability to repetitively trap and release molecules for single-molecular sensing. Finally, this work represents a simple proof-of-concept experiment for single-molecule trapping and detection. The polymer used in this work can be easily extended to other stimuli-responsive polymer systems that are sensitive to humidity, pH and light.

## Methods

### Fabrication of smart plasmonic molecular traps

A freshly prepared 15 nm gold/110 nm silica-coated silicon substrate (size: 4 × 6 mm) was immersed into 200 μl of 2 mM AHT ethanolic solution overnight and then washed with Milli-Q water five times. Subsequently, the substrate was placed into a humidity chamber and a few droplets of water were placed into the chamber to control the humidity. After that, 20 μl of DNA-AuNPs (particle concentration: 7.2 × 10^−11^ M) was placed on the substrate, whereon the substrate was incubated at room temperature for 2 h. The substrate was washed with water five times and dried with a stream of N_2_. Following the AuNP self-assembly, the substrate was subjected to the following sequence of treatments: DTT treatment (200 μl of 0.5 M DTT aqueous solution, overnight), oxygen plasma etching (200 mTorr air, 30 s; Harrick Plasma Cleaning Instrument) and HS-PNIPAM treatment (200 μl of 0.25 M HS-PNIPAM ethanolic solution, overnight). The PNIPAM-coated AuNPs on gold film was then washed with water five times to remove excess HS-PNIPAM molecules.

### SERS activity measurements

Approach 1: a freshly prepared molecular trap was immersed into 200 μl of R6G solution containing 8 mM oxalic acid (R6G concentration: 0.5, 1, 10 and 100 μM, respectively; pH≈2) and then heated to 50 °C. The sample was kept at this temperature for 3 min and then cooled down to 4 °C. Subsequently, the substrate was washed with a cold oxalic acid solution (4 °C) five times to remove excess R6G molecules. This washing process takes only 2–5 min. Thereafter the sample was removed from the cold oxalic acid solution. The sample dried instantaneously during this process. The subsequent SERS measurements were performed in air. To release the analyte, the sample was exposed to a hot oxalic acid solution (50 °C) for 10 min and then washed with the hot oxalic acid five times. Following the final washing step, the sample was taken out from the hot oxalic acid solution and dried immediately. The SERS measurements were conducted in air. To remove the nonspecifically adsorbed R6G, the substrate was subsequently exposed to a mixture of methanol and water (volume ratio=1:1) for 1 h. This process was repeated three times to completely remove nonspecifically adsorbed R6G. After that, the sample was taken out from the mixture and dried, which takes just a few seconds in air. Then the SERS measurements were performed in air. For comparison, a freshly prepared molecular trap was exposed to a cold oxalic acid solution (4 °C, 8 mM) containing 100 μM R6G for 3 min and washed with cold water (4 °C) five times. The substrate was dried immediately on removal from the cold oxalic acid solution and then SERS measurements were performed in air.

Approach 2 (blinking and bianalyte experiments): a freshly prepared molecular trap was immersed into an 8-mM oxalic acid solution (pH≈2) with a temperature of 50 °C. The polymers collapsed at this temperature, forming a denser the polymer shell. This minimizes the nonspecific adsorption of target analytes in the polymer shell. Subsequently, a given volume of a mixture of 10 μM R6G and CV (ratio: 1:1; 50 °C) was added to the oxalic acid solution under vortex mixing to adjust the final concentration of R6G and CV to be 0.5 μM, respectively. The sample was kept at 50 °C for 3 min and then cooled down to 4 °C. Subsequently, the substrate was washed with cold oxalic acid solution (4 °C) five times to remove excess R6G and CV molecules. The substrate was dried in air before the SERS activity measurements. SERS spectra of the smart plasmonic molecular trap were recorded using a Renishaw RM 2,000 Confocal micro-Raman System equipped with a laser at a wavelength of 633 nm (laser power: ∼10 mW; excitation power: ∼3.5 mW; laser spot size: ∼2 μm^2^). All of the Raman spectra were collected by fine focusing a × 50 microscope objective and the data acquisition time was 10 s.

### Simulation

Three-dimensional finite-difference time-domain simulations were performed on a single gold sphere on the gold-coated substrate indicated, enclosed in a domain with a size of 200 × 200 × 400 nm^3^, lined with perfectly matched layers to suppress spurious reflections. The particle–substrate gap and the refractive index of the polymer were adjusted from 0.3 to 1 nm and from 1.2 to 1.5, respectively. The square mesh size was 0.1 nm, which proved to give an acceptable spatial resolution down to the matching nanogap size. The sphere was excited by a plane-wave total-field scattered-field source ranging from 400 to 900 nm, and the total and scattered fields were collected by sets of monitors surrounding the particle and substrate. A three-dimensional monitor was employed to measure the local normalized electric field intensities in the nanogap and integrate them inside the hotspot. The average SERS EF (EF_avg_) is defined by


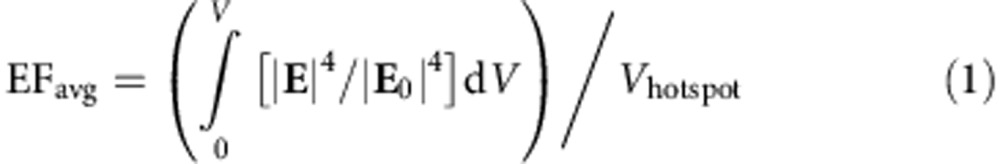


where **E** and **E**_0_ are the local normalized and incident fields, respectively. *V* is the collective volume of all the hotspots evaluated by the integral of all the volume elements with SERS EFs >10^7^.

## Additional information

**How to cite this article:** Zheng, Y. *et al.* Reversible gating of smart plasmonic molecular traps using thermoresponsive polymers for single-molecule detection. *Nat. Commun.* 6:8797 doi: 10.1038/ncomms9797 (2015).

## Supplementary Material

Supplementary Information Supplementary Figures 1-6, Supplementary Tables 1-2, Supplementary Note 1 and Supplementary Methods.
