# Supplementary material for: Reversible gating of smart plasmonic molecular traps using thermoresponsive polymers for single-molecule detection
Source: Nat Commun. 2015 Nov 9;6:8797. doi: 10.1038/ncomms9797 (PMC4667617; doi:10.1038/ncomms9797)
Supplement: Supplementary Information — Supplementary Figures 1-6, Supplementary Tables 1-2, Supplementary Note 1 and Supplementary Methods. [file ncomms9797-s1.pdf]

Supplementary Figures

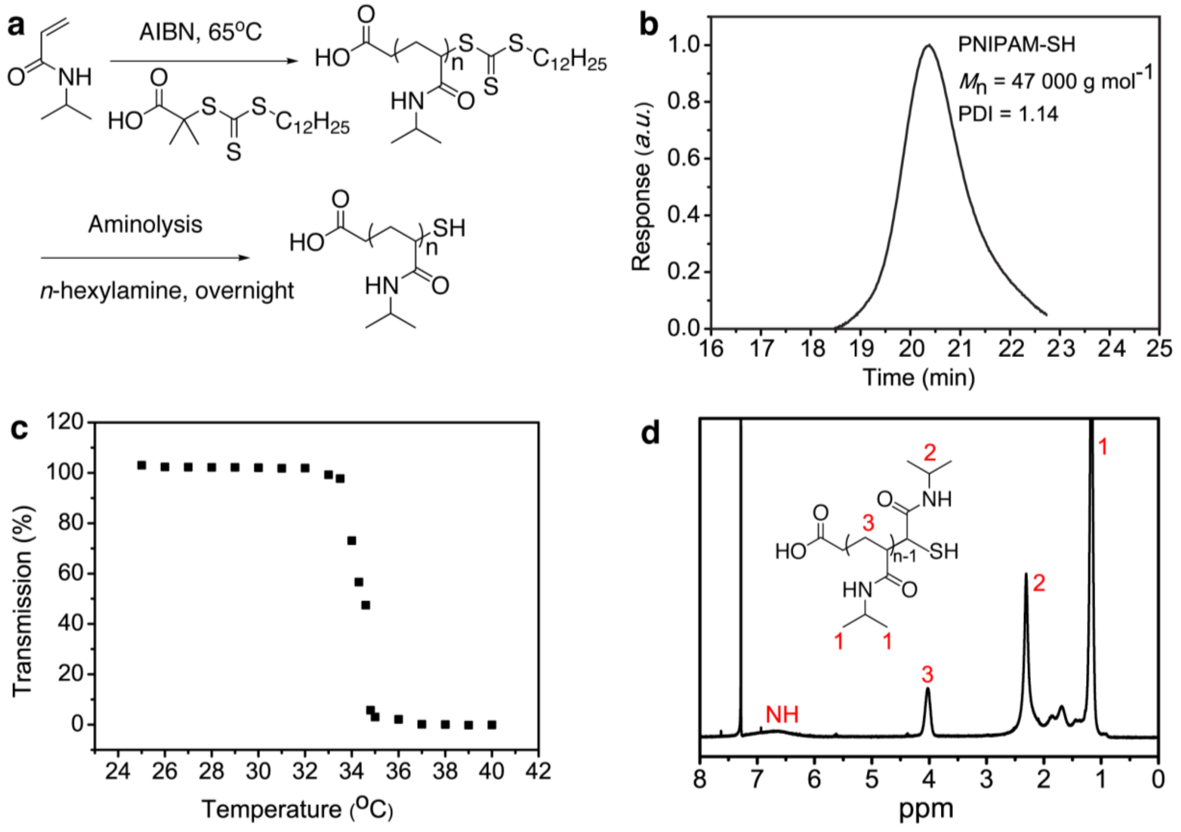

Supplementary Figure 1 | Thiolated thermoresponsive polymer synthesis and characterisation. **a**, Schematic presentation of monothiol poly(*N*-isopropylacrylamide) (HS-PNIPAM) synthesis via RAFT polymerization. **b-d**, Polymer characterisation through Gel Permeation Chromatography (panel **b**), NMR spectrum (panel **c**) and UV-Vis spectrum (panel **d**). The polymer shows a narrow polymer distribution (PDI =1.14) with molecular weight of 47 000 g mol^{-1} (panel **b**) and a thermo responsive behaviour with cloud point at 34.5 °C (panel **c**). ^{1}H NMR (300 MHz, $CDCl_3$), δ (ppm): 6.2-7.1 (broad, single, -NH), 2.36-1.5 (broad, multiple, polymer backbones), 1.25 (broad, single, -CH_3) and $M_{n,NMR}$ 52 000 g mol^{-1} (panel **d**).

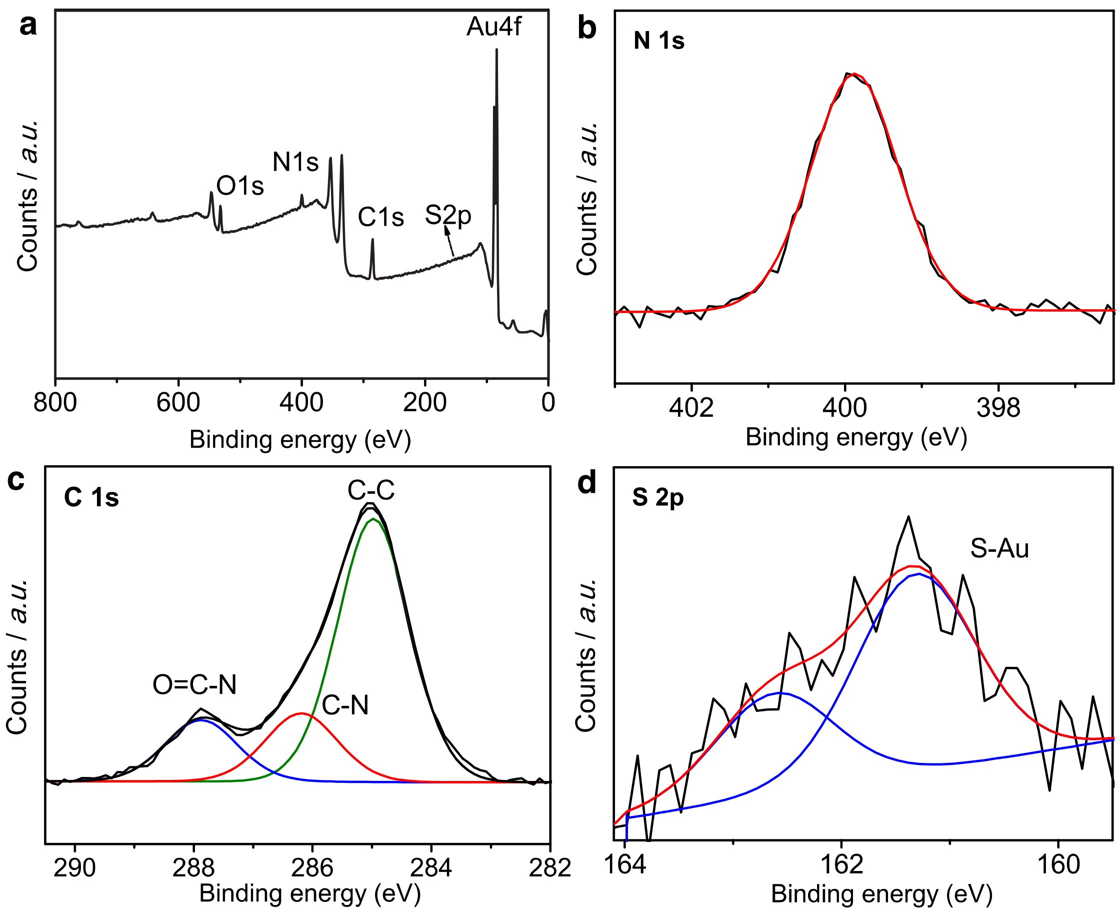

Supplementary Figure 2 | Surface elemental analysis. a, Typical XPS survey spectrum and **b-d**, high-resolution N 1s (panel **b**), C 1s (panel **c**), and S 2p (panel **d**) XPS spectra taken from smart plasmonic molecule traps (i.e. HS-PNIPAM coated AuNPs on the gold film).

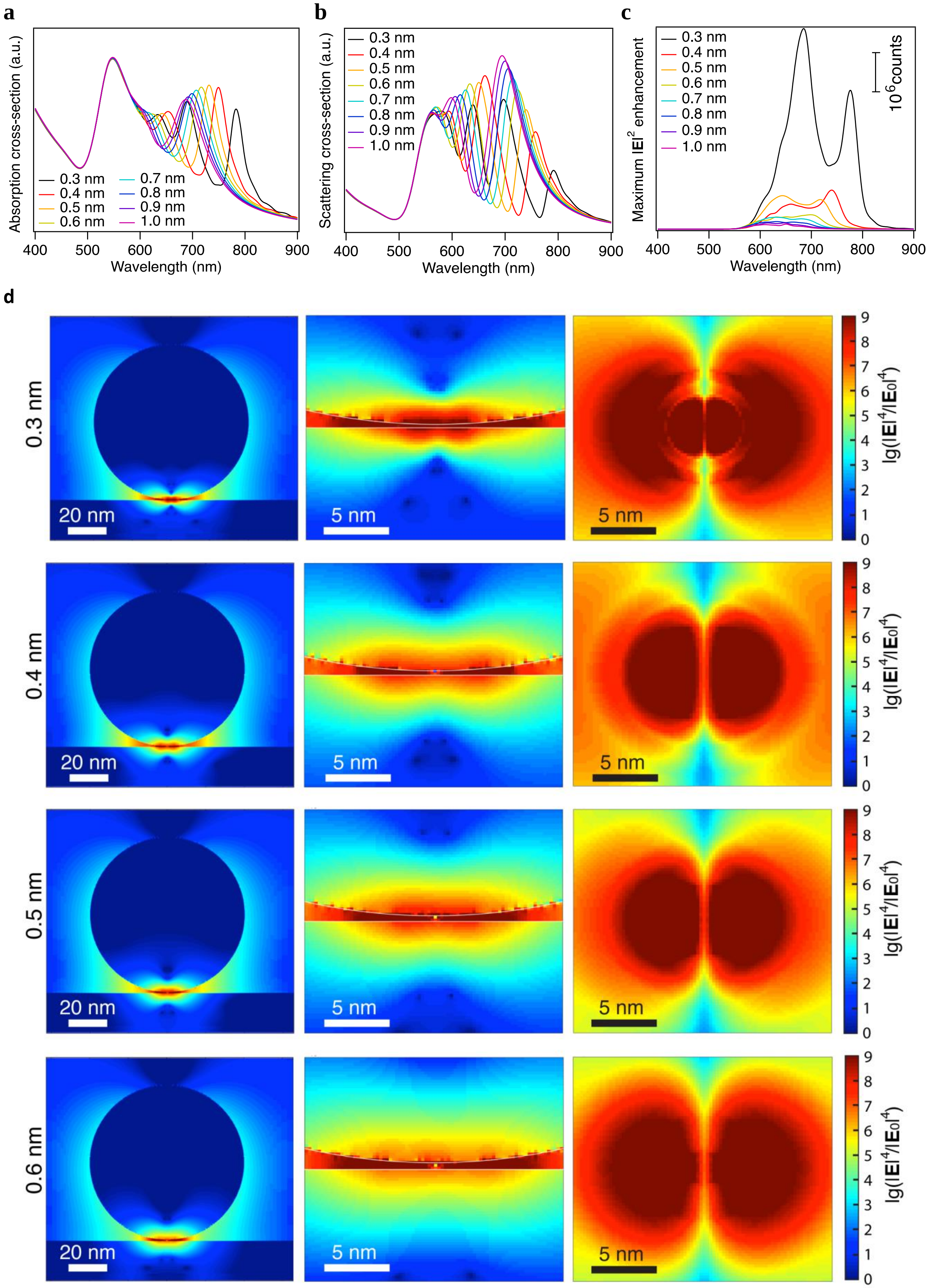
a
Absorption cross-section (a.u.)
Wavelength (nm)
0.3 nm
0.4 nm
0.5 nm
0.6 nm
0.7 nm
0.8 nm
0.9 nm
1.0 nm
b
Scattering cross-section (a.u.)
Wavelength (nm)
c
Maximum |E|^2 enhancement
Wavelength (nm)
10^{6}counts
d
0.3 nm
0.4 nm
0.5 nm
0.6 nm
20 nm
5 nm
lg(|E|^4/|E$_0$|^4)

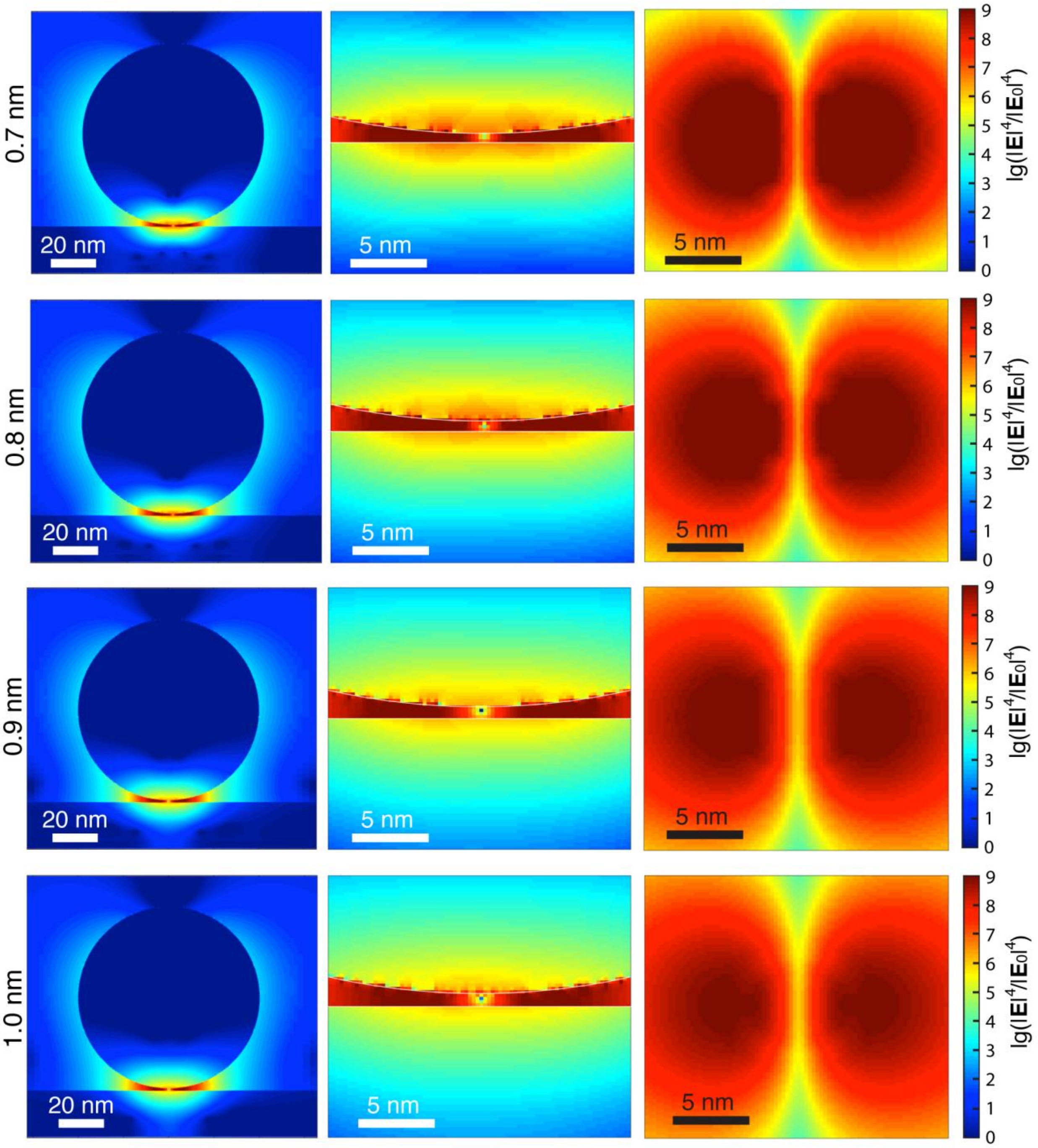

Supplementary Figure 3 | Simulated results. a, Simulated absorption spectra, **b**, scattering spectra, and **c**, maximum electric field enhancement spectra as a function of particle-substrate gap (w). **d**, Spatial $|\mathbf{E}|^4/|\mathbf{E}_0|^4$ -distributions at a 80 nm AuNP-15 nm Au film junction as a function of w (left column) and close-up of $|\mathbf{E}|^4/|\mathbf{E}_0|^4$ -distributions at the particle-substrate gap along the planes vertical (xz, middle column) and horizontal (xy, right column) to the sample plane (xy), respectively. The x-, y-, z-axes unit for all pictures is meter.

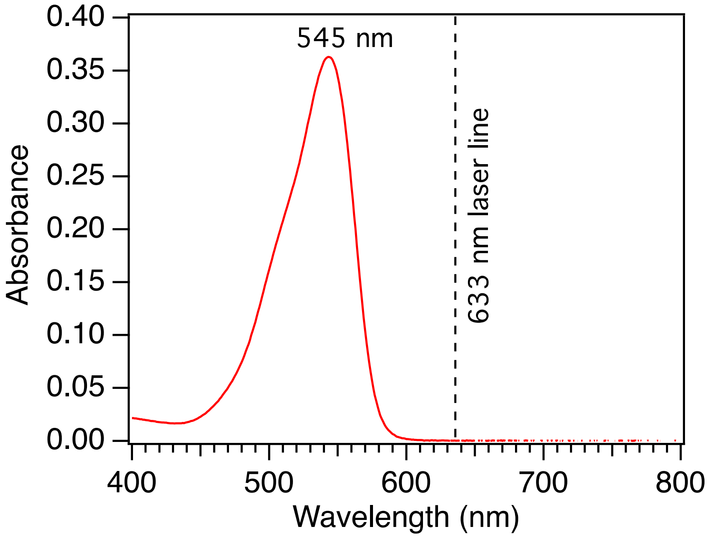

Supplementary Figure 4 | Absorption spectrum of a 10 µM rhodamine 6G aqueous solution. The 633 nm laser line used for Raman excitation is shown in the picture.

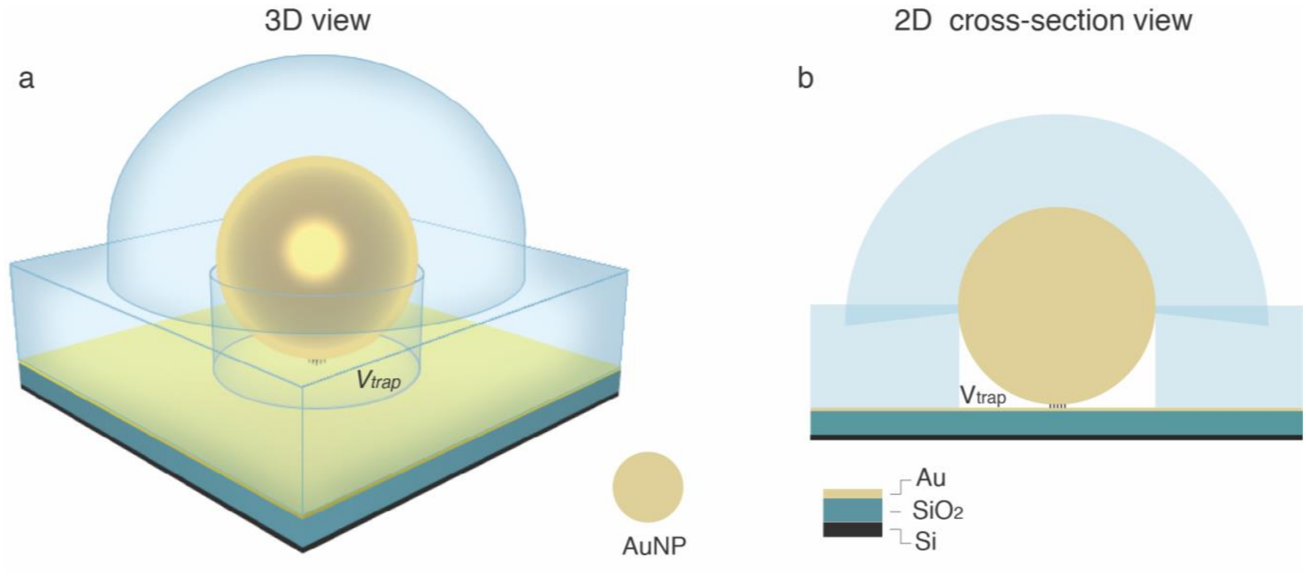

Supplementary Figure 5 | Schematic presentation of the smart molecular trap. a, 3D view and **b,** 2D cross-section view of smart molecular trap with gate closed (i.e. the polymer shell is in a expanded conformation). V_{trap} is the volume of a single molecular trap.

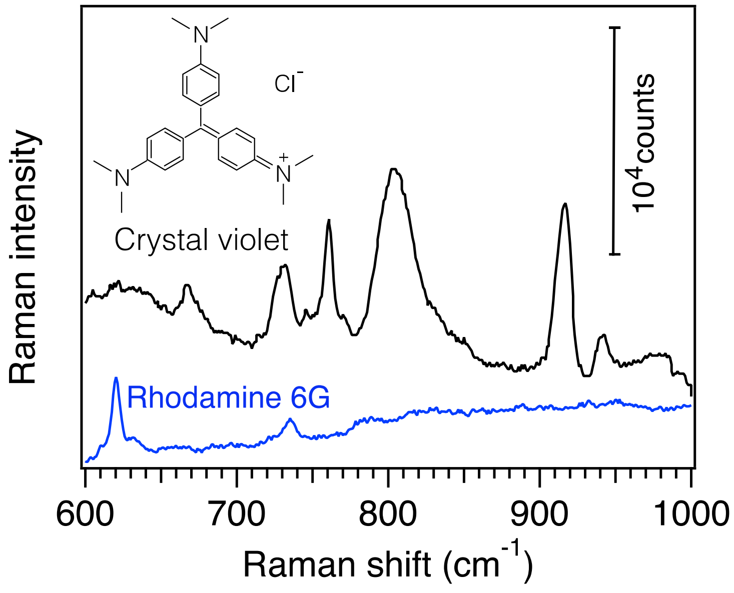

Supplementary Figure 6 | SERS spectra of analytes from from the molecular traps. The analytes investigated here are rhodamine 6G (blue curve) and crystal violet (black curve), respectively. The insert is the molecular structure of crystal violet. For all samples, analyte loading concentration: 10 μM, λ_{ex} = 633 nm, acquisition time = 10 s and laser spot size ≈ 2 μm^2. The excitation power are ~3.5 and ~0.35 mW for rhodamine 6G and crystal violet, respectively.

Supplementary Tables

Supplementary Table 1 | Elemental percentages of the smart plasmonic molecular traps.

**Peak**	**Atomic %**
C 1s (C-C)	39.2%
C 1s (C-N)	9.2%
C 1s (O=C-N)	10.2%
N 1s	8.1%
S 2p	0.5%

Theoretical C-C / C-N / O=C-N for poly(*N*-isopropylacrylamide) = 4:1:1

Experimental C-C / C-N / O=C-N: 4.3: 1: 1.1

Theoretical N/C ratio for poly(*N*-isopropylacrylamide): 1:6

Experimental N/C ratio: 1:7.2

Supplementary Table 2 | Summary of hotspot volume and average SERS enhancement calculated using the SERS enhancement boundary criterion of 10^7.

w (nm)	Max $|\mathbf{E}|^2$ enh. (norm.) at 621 cm^{-1}	Integral of intens. squared	Volume of hot spot (m^3)	Average SERS enhancement
0.3	3.3×10^{6}	6.4×10^{-16}	3.8×10^{-26}	1.67×10^{10}
0.4	6.4×10^{5}	1.4×10^{-16}	2.4×10^{-26}	5.84×10^{9}
0.5	7.7×10^{5}	1.4×10^{-16}	2.9×10^{-26}	5.06×10^{9}
0.6	3.1×10^{5}	5.9×10^{-17}	3.8×10^{-26}	1.55×10^{9}
0.7	2.5×10^{5}	4.9×10^{-17}	4.8×10^{-26}	1.02×10^{9}
0.8	1.8×10^{5}	3.2×10^{-17}	5.6×10^{-26}	5.74×10^{8}
0.9	1.2×10^{5}	2.2×10^{-17}	6.2×10^{-26}	3.54×10^{8}
1.0	9.7×10^{4}	1.4×10^{-17}	6.5×10^{-26}	2.26×10^{8}

Supplementary Notes

Supplementary Note 1: Calculation of numbers of molecules trapped per laser beam spot

Parameters used for calculation: analyte concentration (C = 1 μM), AuNP radius (r = 40 nm), particle density on the gold/silica bilayer coated silicon substrate (ρ = 14 particles/μm^2) and laser spot size (S = 2 μm^2). The volume of a single molecular trap (V_{trap}) is calculated based on the scheme shown in Supplementary Fig. 5.

The volume of a single molecular trap (V_{trap}):

$$V_{trap} = \pi r^2 \times r - 2/3\ \pi r^3$$

$$= 1/3\ \pi r^3$$

$$= 6.7 \times 10^4\ (nm^3)$$

The total volume of all molecular traps (V) per laser beam spot:

$$V = \rho \times S \times V_{trap}$$

$$= 1.88 \times 10^6\ (nm^3)$$

Number of analyte molecules trapped per laser beam spot (N):

$$N = C \times V$$

$$= 1.13\ (\text{molecules})$$

Therefore, when the analyte concentration is 1 μM, only about 1 molecule is trapped in the hot spots under laser beam irradiation.

Supplementary Methods

Synthesis of thiolated poly(*N*-isopropyl acrylamide) (HS-PNIPAM). In a typical procedure, *N*-isopropyl acrylamide (NIPAM; recrystallised from hexane) (2.5 g, 22.1 mmol), Reversible Addition Fragmentation chain Transfer (RAFT) agent 2-(dodecylthiocarbonothioylthio)-2-methyl propionic acid (15.2 mg, 0.041 mmol), 2,2'-azobisisobutyronitrile (AIBN; recrystallised from methanol) (1 mg, 6×10^{-3} mmol) and 1,4-dioxane (8 ml) were all sealed in 20 mL vial. After purging for 30 min, the solution was heated to 65 °C for a predetermined time. The reaction solution was diluted with tetrhahydrofuran (THF) and the polymeric product (i.e. PNIPAM-RAFT) was precipitated into diethyl ether three times and dried under vacuum. To yield HS-PNIPAM, PNIPAM-RAFT (1 g, 0.021 mmol) and n-hexylamine (10.7 mg, 0.1 mmol) were dissolved in ethanol (5 ml), which was then degassed by purging with N_2 for 10 min. The solution was stirred under N_2 at room temperature overnight. The crude product was dried under air and redissolved in THF before precipitation into cold hexane three times. The resulting white powder (HS-PNIPAM) was then collected and dried under vacuum.

Synthesis of DNA-functionalized gold nanoparticles (DNA-AuNPs). Commercially available spherical AuNPs were functionalized with thiolated DNA in the presence of polyoxyethylene (20) sorbitan monolaurate (Tween 20). 1 mL of citrate stabilized AuNP solution (diameter: 80 nm, particle concentration: 1.8×10^{-11} M; Ted Pella) was concentrated to 50 µL by centrifugation (Eppendorf 5415R centrifuge; 3000 rpm, 40 min). Volumes of 5 µL of 2.0% Tween 20, 20 µL of 100 µM monothiol DNA solution (sequence: 5′-[HS]-T15-TAA TCA GGG TCA TAA-3′, Fidelity Systems Inc.) and 30 µL of 0.1 M phosphate buffer (pH = 7.0) were successively added into the AuNP solution. The mixture was incubated at room temperature for 1 h. The NaCl concentration of the mixture was then increased to 0.02 M using 2 M NaCl and incubated for 20 min. This process was repeated four times, in increments of 0.02 M NaCl, and then every 0.1 M NaCl increment thereafter, until a concentration of 0.5 M NaCl was reached. After that, the mixture was incubated at 4 °C overnight. The DNA-AuNPs were washed five times with Milli-Q water and then redispersed in 100 µL of Milli-Q water.

Fabrication of gold/silica bilayer coated silicon substrate. Gold/silica bilayer coated silicon substrate was fabricated by deposition of 15 nm gold onto a silica-coated silicon wafer using self-assembled layer of 3-mercaptopropyltrimethoxysilane (MPTES) as an adhesion layer. In a typical procedure, a commercially available silica-coated silicon wafer (silica thickness: 110 nm, purchased from Universitywafer) was cleaned with piranha solution (A mixture of concentrated sulfuric acid to 30% hydrogen peroxide solution with volume ratio of 3:1). Subsequently, the wafer was modified with MPTES by immersion into a mixture of MPTES, water, and ethanol at volume ratio of 2:3:95 for 1 h. The wafer was then washed with ethanol three times. The gold deposition was conducted on an Intlvac Nanochrome II electron beam evaporation system at the Melbourne Center for Nanofabrication (MCN). The deposition rate was controlled to be 0.012 nm s^{-1}. Following the gold deposition, the wafer was cut into small pieces with size of 4 mm × 6 mm.

Characterisation. The molecular weight and polydispersity of HS-PNIPAM were characterized by size exclusion chromatography (SEC) in dimethyl acetamide. SEC analysis were performed at 40 °C (flow rate = 1 mL min^{-1}) using a Shimadzu modular system equipped with RID-10A RI detector. Molecular weight separation was achieved via a column set comprising a PL 5.0 mm bead-size guard column and four phenomenex PHENOLGEL columns (300 × 7.8 mm; 5 µm; 10^2, 10^3, 10^4,

and 10^6 Å). A calibration curve was generated with commercial linear polystyrene standards ranging from 500 to 10^6 g mol^{-1}. The Mark-Houwink parameters used to determine the molecular weight of the HS-PNIPAM were $K = 10^{-(4.24 \pm 0.42)}$ dL g^{-1} and $\alpha = 0.78 \pm 0.09$. Polymerization conversion was determined by nuclear magnetic resonance (NMR) spectroscopy. 1H NMR spectra were recorded using a Bruker ACF300 (300MHz) spectrometer employing $CDCl_3$ as the solvent. SEM images of the self-assembled AuNPs were taken with a JEOL 7001F field emission SEM. And the absorption spectra of the self-assembled AuNPs were recorded using a Cary 60 UV-vis spectrometer.
